# A measles IgM rapid diagnostic test to address challenges with national measles surveillance and response in Malaysia

**DOI:** 10.1371/journal.pone.0298730

**Published:** 2024-03-14

**Authors:** A’aisah Senin, Noorliza M. Noordin, Jamiatul A. M. Sani, Diana Mahat, Morgane Donadel, Heather M. Scobie, Aziyati Omar, Yu K. Chem, Mohamad I. Zahari, Fatanah Ismail, Rozita A. Rahman, Hani M. Hussin, Sengol Selvanesan, Zirwatul A. Aziz, W. N. Afiza W. M. Arifin, Rehan S. A. Bakar, Norhayati Rusli, M. Hanif Zailani, Paul Soo, Ying-Ru Lo, Varja Grabovac, Paul A. Rota, Mick N. Mulders, David Featherstone, Lenesha Warrener, David W. Brown

**Affiliations:** 1 Disease Control Division, Ministry of Health Malaysia, Kuala Lumpur, Malaysia; 2 Global Immunization Division, Centers for Disease Control and Prevention, Atlanta, Georgia, United States of America; 3 National Public Health Laboratory, Ministry of Health Malaysia, Kuala Lumpur, Malaysia; 4 Family Health Development Division, Ministry of Health Malaysia, Kuala Lumpur, Malaysia; 5 Office of the World Health Organization Representative to Malaysia, Brunei Darussalam and Singapore, Cyberjaya, Malaysia; 6 World Health Organization Regional Office for the Western Pacific, Manila, Philippines; 7 Division of Viral Diseases, Centers for Disease Control and Prevention, Atlanta, GA, United States of America; 8 Department of Immunization, Vaccines and Biologicals, World Health Organization, Geneva, Switzerland; 9 Consultant Scientists Ltd, Auckland, NewZealand; 10 Public Health Microbiology Division, United Kingdom Health Security Agency (UKHSA), London, United Kingdom; 11 Laboratório de Vírus Respiratórios e do Sarampo, Instituto Oswaldo Cruz/Fiocruz, Rio de Janeiro, Rio de Janeiro, Brazil; CEA, FRANCE

## Abstract

**Introduction:**

A lateral flow rapid diagnostic test (RDT) enables detection of measles specific immunoglobulin M (IgM) antibody in serum, capillary blood, and oral fluid with accuracy consistent with enzyme immunoassay (EIA). The objectives of the study were: 1) to assess measles RDT inter-reader agreement between two clinic staff; 2) to assess the sensitivity and specificity of the measles RDT relative to standard surveillance testing in a low transmission setting; 3) to evaluate the knowledge, attitudes, and practices of staff in clinics using the RDT; and 4) to assess the impact of RDT testing on the measles public health response in Malaysia.

**Materials and methods:**

The clinic-based prospective evaluation included all suspected measles cases captured by routine measles surveillance at 34 purposely selected clinics in 15 health districts in Malaysia between September 2019 and June 2020, following day-long regional trainings on RDT use. Following informed consent, four specimens were collected from each suspected case, including those routinely collected for standard surveillance [serum for EIA and throat swabs for quantitative reverse transcriptase polymerase chain reaction (RT-qPCR)] together with capillary blood and oral fluid tested with RDTs during the study. RDT impact was evaluated by comparing the rapidity of measles public health response between the pre-RDT implementation (December 2018 to August 2019) and RDT implementation periods (September 2019 to June 2020). To assess knowledge, attitudes, and practices of RDT use, staff involved in the public health management of measles at the selected sites were surveyed.

**Results:**

Among the 436 suspect cases, agreement of direct visual readings of measles RDT devices between two health clinic staff was 99% for capillary blood (k = 0.94) and 97% for oral fluid (k = 0.90) specimens. Of the total, 45 (10%) were positive by measles IgM EIA (n = 44, including five also positive by RT-qPCR) or RT-qPCR only (n = 1), and 38 were positive by RDT (using either capillary blood or oral fluid). Using measles IgM EIA or RT-qPCR as reference, RDT sensitivity using capillary blood was 43% (95% CI: 30%–58%) and specificity was 98% (95% CI: 96%–99%); using oral fluid, sensitivity (26%, 95% CI: 15%–40%) and specificity (97%, 95% CI: 94%–98%) were lower. Nine months after training, RDT knowledge was high among staff involved with the public health management of measles (average quiz score of 80%) and was highest among those who received formal training (88%), followed by those trained during supervisory visits (83%). During the RDT implementation period, the number of days from case confirmation until initiation of public response decreased by about 5 days.

**Conclusion:**

The measles IgM RDT shows >95% inter-reader agreement, high retention of RDT knowledge, and a more rapid public health response. However, despite ≥95% RDT specificity using capillary blood or oral fluid, RDT sensitivity was <45%. Higher-powered studies using highly specific IgM assays and systematic RT-qPCR for case confirmation are needed to establish the role of RDT in measles elimination settings.

## Introduction

Measles is a severe and potentially fatal vaccine-preventable disease that is targeted for elimination in all World Health Organization (WHO) regions [1]. In the Western Pacific Region, many countries have achieved or are approaching measles elimination; however, surveillance including outbreak investigation in remote areas (e.g., islands) remains a logistical challenge [2]. Use of a measles rapid diagnostic test (RDT) would facilitate rapid case confirmation and public health response, even in hard-to-reach areas [3, 4].

One lateral flow RDT based on an antibody capture format for measles immunoglobulin M (IgM) has been described, which enables the detection of measles-specific IgM antibodies in both serum and oral fluid specimens in less than 30 minutes [5, 6]. The measles RDT had high (>90%) sensitivity and specificity with serum and oral fluid compared to a reference enzyme immunoassay (EIA) [5, 7]. Measles virus ribonucleic acid (RNA) was able to be detected by quantitative reverse transcriptase polymerase chain reaction (RT-qPCR) and genotyped after being recovered from the specimen pad of used IgM positive RDTs that had been stored dry 5 weeks at 20–25°C [5]. This prototype RDT was redesigned as a cassette-housed assay to ensure ease of handling and robustness for field testing [7], subsequently modified for use with capillary blood using the ASSURED criteria [8] and produced under subcontract manufacture. A trial batch of this redesigned RDT was evaluated in this study.

In Malaysia, case-based measles surveillance involves case detection and diagnosis, case notification, case investigation, and public health response. Suspected measles cases are detected at hospitals, public and private health centres, and community clinics. Malaysia’s measles surveillance system relies on Global Measles Rubella Laboratory Network (GMRLN)-accredited national laboratories and four subnational laboratories (not yet WHO-accredited but participating in global external quality assessment). Measles cases are laboratory-confirmed based on positive IgM serology, measles viral isolation, or real-time RT-qPCR viral detection; rubella surveillance is integrated with measles surveillance including rubella testing of specimens testing negative or indeterminate for measles IgM. Public health response includes contact investigation, active case detection, and mop-up vaccination within one kilometer of the case within 72 hours of laboratory confirmation. Measles incidence in Malaysia reached 62.1 per million population in 2018 [9]. Despite Malaysia’s progress towards measles elimination, having achieved >95% coverage with two doses of measles and rubella (MR)-containing vaccine, the country still reported a high number of measles cases (>1,000 yearly during 2015–2019) that needed confirmation and response [2, 10].

Addressing a priority within the Measles & Rubella Initiative Laboratory and Surveillance Research Agenda [3], the aim of this study was to assess the feasibility and potential impact of measles RDT use as part of the national surveillance program in Malaysia. The objectives included: 1) to assess measles RDT reading agreement between two clinic staff; 2) to assess the performance of the measles IgM RDT relative to measles IgM EIA and RT-qPCR testing in a low transmission setting; 3) to evaluate the acceptability, knowledge, attitudes, and practices (KAP) of staff in clinics using the measles RDT; and 4) to assess the impact of RDT testing on the rapidity of measles public health response.

## Materials and methods

### Study design and population

This was a clinic-based, prospective evaluation of the feasibility of integrating measles RDT use into national surveillance and the potential impact on measles surveillance and outbreak response. The study population included all suspected measles cases (i.e., any person with a history of fever and maculopapular rash with any one of cough, coryza, or conjunctivitis) captured by routine measles surveillance at 34 selected clinics (~1.5% of the total number of clinics in Malaysia) spread across 15 health districts during September 1, 2019, to June 30, 2020. Health clinics were purposefully selected based on high numbers of reported confirmed measles cases within the prior 12 months; consideration was given to logistical feasibility (i.e., geographic location and transport logistics) in terms of inclusion of clinics for the study. Following informed consent, four specimens were collected from each suspected case, including two used for standard surveillance (serum for EIA and throat swabs for RT-qPCR) together with capillary blood and oral fluid tested with RDTs during the study. All RDT results were interpreted visually at 20 minutes by two independent readers at the clinic (see Laboratory Testing for further details). A sample size of 450 suspected measles cases was chosen based on what would be logistically feasible and was estimated to give a power of >95% to detect “substantial” agreement [Cohen’s kappa (k) > 0.7; alternative = 0.4] between two readers with 10% positive tests and α = 0.05. We also assessed the sensitivity, specificity, and positive predictive value (PPV) of the measles RDT compared with measles IgM EIA or RT-qPCR, though the study was not specifically powered for this objective.

The impact evaluation involved a “pre-post” implementation design to evaluate public health response indicators for the clinics selected to use the RDT. The pre-RDT implementation period was defined as a period without RDT use (December 2, 2018, to August 30, 2019), while the RDT implementation period was defined as the period with RDT use (September 1, 2019 to June 30, 2020). Identical data collection forms, which included case investigation and public health response data, were used during the pre- and post- RDT implementation periods. The implementation included eight regional trainings with structured day-long agendas of didactic and practical sessions on specimen processing and performing RDTs during August 2019; a total of 238 staff were trained during these formal sessions. The trainings were conducted by UK Health Security Agency (UKHSA, formerly Public Health England) and US Centers for Disease Control and Prevention (US-CDC) laboratory and epidemiology staff jointly with the Malaysian Ministry of Health (MOH) team running the project, followed soon after by cascade trainings performed by trained local staff. Following the training, RDT testing was established in the selected clinics, with members of the Malaysian national MOH team performing periodic remote and in-person monitoring and supervision. For this part of the evaluation, we estimated that for 45 confirmed measles cases (10% of 450 suspected cases), statistical power to detect a difference in two sample means (2-sided test) would be 89%, assuming a baseline of 10 days response time and a response time of 6 days during RDT implementation, with a standard deviation of 6 days.

To assess acceptability and KAPs of measles RDT use, staff involved in the public health management of measles at all the selected sites (e.g., clinicians, health clinic laboratorians, district epidemiologist, and district public health inspectors) were asked to complete an electronic survey (SurveyMonkey, San Mateo, CA) at the end of the study, between May 4 and June 15, 2020. The survey included socio-demographic information and KAP questions regarding collection and processing of specimens, performing RDTs, reporting results, and the programmatic impact of RDT. We compared the quiz score from the knowledge section of the KAP survey to the score from a post-training knowledge quiz (SurveyMonkey, San Mateo, CA) with the same questions given to trainees at the end of the formal training in August 2019 (9 months earlier).

### Measles IgM RDT

The measles IgM RDT is an immunochromatographic assay enclosed in a plastic cassette. Components and the principle of the assay have been described previously [7]. A trial batch of measles IgM RDTs was prepared under contract manufacture, according to the specifications of the UKHSA. The RDTs were provided in individual foil-mylar pouches, each containing a desiccant sachet and stored at room temperature (22–32°C). Extraction buffer of phosphate buffered saline (pH 7.2), 10% heat-treated fetal calf serum, 0.5% Tween® 20, with Gentamicin (250μg/ml) and Amphotericin B (0.5μg/ml) was stored at 2–8°C.

### Laboratory testing

A flow chart describing the organization of specimen collection, testing and reporting is given in S1 Fig. Following enrollment, capillary blood and oral fluid specimens were collected, in addition to two standard surveillance specimens, serum from whole blood, and a throat swab. For the measles IgM RDTs, capillary blood samples were collected using lancets, and blood was diluted 1:54 in extraction buffer, before approximately 100μl was added to RDT for testing. Oral fluid specimens were collected using the Oralight device (UK Health Security Agency [formerly Public Health England], London, United Kingdom), eluted with 1.5ml of extraction buffer, and four drops dispensed directly into the RDT from the Oralight device dropper. The procedure and training materials stated that RDTs were to be incubated for 20 minutes, read by two independent readers at the clinic, and interpreted as: positive if there was a visible “test” line, negative if there was no “test” line, or indeterminate if readers found it difficult to see a “test” line. RDTs were interpreted as valid if a “control” line was visible and as invalid if the “control” line was not visible. The clinic staff placed each used dried RDT in an individual plastic bag with a desiccant sachet and the case investigation form attached; this was shipped at 2–8°C to the National Public Health Laboratory (NPHL) together with the separated serum and throat swab.

During the pre-RDT implementation period, sera from suspected cases were tested for measles IgM using the Enzygnost® measles IgM indirect EIA (Siemens Healthcare GmbH, Marburg, Germany), while in the RDT implementation period, sera were tested using the Virion/Serion measles IgM indirect EIA (GmbH, Würzburg, Germany), per manufacturer’s instructions. Sera from measles suspected cases were also tested for rubella IgM using the Virion/Serion rubella IgM EIA (GmbH, Würzburg, Germany) or the Enzygnost® rubella IgM EIA (Siemens Healthcare GmbH, Marburg, Germany). The difference in tests used was related to global supply issues. Since false positive IgM results may be found in a higher proportion than true positives in a low incidence setting close to elimination, sera from suspected cases with discordant measles IgM results (positive in either the EIA or RDT but not both) were also tested in the Microimmune measles IgM capture EIA (Microimmune Ltd, Hounslow, England). RNA from throat swabs and the specimen pad from each RDT used for testing oral fluid and capillary blood specimens was extracted using the QIAamp Viral RNA Mini Kit (Qiagen, Hilden, Germany) and tested by N-gene RT-qPCR for measles RNA using an established assay and by human RNAse P-gene RT-qPCR as a control for measles-negative specimens [5, 11]. Because N-gene RNA contamination of the RDTs from the expression system used to prepare N antigen is possible, RNA extracted from used RDTs was tested using an established measles H-gene RT-qPCR [5]. RT-qPCR products from positive measles specimens were purified using QIAquick PCR Purification Kit (Qiagen, Germany), followed by cycle sequencing at a commercial facility using the BigDye Terminator Cycle Sequencing kit (Applied Biosystems, USA) and Sanger sequencing of the N-gene [7].

### Data analysis

Data were analyzed using RStudio (Boston, MA) and SAS version 9.4 (SAS Institute, Cary, NC). We calculated Cohen’s kappa coefficient (k) to assess inter-rater reliability in visual RDT readings between two clinic staff. The sensitivity, specificity, and PPV estimates of the RDT compared with standard surveillance testing were calculated with their corresponding 95% Wilson confidence intervals (95% CI). RDT results from capillary blood and oral fluid were compared using two definitions of “true positive” towards the aim of increasing the specificity of case classification in a low-incidence setting: 1) positive with measles IgM EIA (Virion/Serion) or RT-qPCR, or 2) positive with measles IgM EIA (Virion/Serion AND Microimmune) or RT-qPCR. A measles vaccine-associated reaction was defined per WHO measles surveillance guidelines [12]; a suspected case that meets all five of the following criteria: (1) the patient had a rash illness, but did not have cough or other respiratory symptoms related to the rash; (2) the rash began 7–14 days after vaccination with a measles-containing vaccine; (3) the blood specimen, which was positive for measles IgM, was collected 8–56 days after vaccination; (4) a thorough field investigation did not identify any secondary cases; (5) field and laboratory investigations failed to identify other causes, or genotype A was isolated from the suspected case (genotype A is only vaccine-related and does not occur as wild-type infection). In Malaysia, the public health response to a measles vaccine-associated reaction remains the same as for other measles case definitions. A sensitivity analysis was also conducted where indeterminate and discordant visual readings were considered as positive. For the KAP, the frequency of responses was summarized in a descriptive analysis. For the impact evaluation, we compared the mean number of days for different public health response activities in the pre-RDT and RDT implementation periods using Wilcoxon two-sample tests.

### Ethics

This study was determined to be a public health program evaluation activity according to the U.S. CDC’s and WHO Western Pacific Regional Office’s human subjects’ procedures. It was approved by the Malaysian Medical Research & Ethics Committee (Approval no. NMRR- 18-2165-43312) and the National Medical Research Registration of Ministry of Health Malaysia (S1 File). Information about the study was provided verbally and in writing to participants, and informed consent was obtained from all participants ≥18 years or caretakers of children. An assent form was also collected from children aged 7–17 years.

## Results

A total of 436 suspected cases provided consent and were enrolled in the evaluation. Of those, 326 (75%) were aged 2 years or younger. All suspected cases had capillary blood and oral fluid specimens collected and tested by RDT, and most had serum specimens tested by measles EIA (n = 434); among the two cases without serum specimens, both had a throat swab collected and tested by RT-qPCR. Eighteen cases were not tested by rubella EIA. Dates of serum collection and rash onset were available for 397 (91%) of cases. For cases with both onset and sample collection dates, most (96%) serum specimens were collected within 7 days of rash onset, and 79% were collected within 3 days. Twelve (3%) cases had no throat swab specimen collected (or insufficient quality); another 41 (9%) did not have RT-qPCR results, mostly (95%) because specimen collection was ≥4 days after rash onset.

### RDT reader agreement

Among the 436 suspected measles cases, 38 (9%) cases overall were positive by RDTs read by health clinic staff at the point-of-care; of these, 15 were positive with capillary blood alone, 11 were positive with oral fluid alone, and 12 were positive with both. The overall agreement of direct visual readings of the RDT device between the two health clinic staff was 99% for capillary blood (k = 0.94) and 97% for oral fluid (k = 0.90) (Table 1).

**Table 1:** Agreement between direct visual readings of measles rapid diagnostic test (RDT) results by clinic staff.

	Direct visual readings by two clinic staff	
Specimen for RDT testing	Capillary blood (n = 436)	Oral fluid (n = 436)
**Concordant reading, n (%)**	430 (99)	423 (97)
**Positive**	27 (6)	23 (5)
**Negative**	382 (88)	361 (83)
**Indeterminate**	15 (3)	21 (5)
**Invalid**	6 (1)	18 (6)
**Discordant reading, n (%)**	6 (1)	13 (3)
**Cohen’s kappa (k)**	0.94	0.90

### Sensitivity, specificity, and PPV of the measles RDT

Among the 436 suspected measles cases, 45 (10%) cases were positive by measles IgM EIA (n = 44; five of these were also positive by RT-qPCR) or RT-qPCR only (n = 1). Of those, 25 (56%) measles IgM-positive serum specimens had been collected 8─56 days after MR vaccination, and 5 of these were confirmed as vaccine-associated reactions. Of the 45 measles positive cases, 16 (36%) were also positive by rubella IgM EIA; of these, 14 (88%) were collected 8─56 days after receiving MR-containing vaccine. Compared with measles IgM EIA Virion/Serion or RT-qPCR as reference, RDT sensitivity using capillary blood and concordant visual readings at clinics was 43% (95% CI: 30%–58%), specificity was 98% (95% CI: 96%–99%), and PPV was 70% (95% CI: 52%–84%) (Table 2 and S1 Table). Sensitivity (26%, 95% CI: 15%–40%), specificity (97%, 95% CI: 94%–98%), and PPV (48%, 95% CI: 29%–67%) were lower using oral fluid.

**Table 2:** Sensitivity, specificity, and positive predictive value (PPV) of measles IgM rapid diagnostic test (RDT) results using capillary blood and oral fluid compared with reference testing*.

	RDT using capillary blood		RDT using oral fluid	
	Reference test			
Indicator and treatment of indeterminate results	Measles IgM EIA Virion/Serion or RT-qPCR	Measles IgM EIA Virion/Serion and Measles IgM EIA Microimmune or RT-qPCR	Measles IgM EIA Virion/Serion or RT-qPCR	Measles IgM EIA Virion/Serion and Measles IgM EIA Microimmune or RT-qPCR
Indeterminate RDT results treated as negatives				
% Sensitivity (95% CI)	43 (30–58)	57 (39–73)	26 (15–40)	38 (23–56)
% Specificity (95% CI)	98 (96–99)	98 (95–99)	97 (94–98)	97 (95–98)
% PPV (95% CI)	70 (52–84)	63 (44–78)	48 (29–67)	48 (29–67)
Indeterminate RDT results treated as positives				
% Sensitivity (95% CI)	61 (47–74)	73 (56–86)	47 (33–61)	55 (38–72)
% Specificity (95% CI)	95 (93–97)	94 (92–96)	92 (89–94)	91 (88–94)
% PPV (95% CI)	60 (45–73)	49 (35–63)	40 (28–54)	32 (21–46)

95% CI: 95% confidence interval; EIA: enzyme immunoassay; PPV: positive predictive value; RDT: rapid diagnostic test; RT-qPCR: quantitative reverse transcriptase polymerase chain reaction

* RDT results from capillary blood and oral fluid were compared to reference test results using two definitions of “true positive” towards the aim of increasing the specificity of case classification in a low-incidence setting: 1) positive with measles IgM EIA (Virion/Serion) or RT-qPCR, or 2) positive with measles IgM EIA (Virion/Serion AND Microimmune) or RT-qPCR. Virion/Serion is an indirect EIA and Microimmune is a capture EIA with higher specificity. Indeterminate RDT results were treated as negative in the primary analysis, or positive in a sensitivity analysis, as indicated.

Sera from 109 suspected cases with discordant RDT and Virion/Serion measles EIA results or indeterminate EIA results (n = 6) were also tested by Microimmune IgM EIA. Among these, 29 (27%) cases had concordant IgM-positive Virion/Serion and Microimmune EIA results, 49 (45%) had concordant negative EIA results, and 31 (28%) had discordant results by either EIA (14 were IgM-positive only using Virion/Serion and eight were positive only using Microimmune). All six of the indeterminate results by Virion/Serion EIA were negative by Microimmune EIA. Of the 29 cases with concordant IgM-positive specimens, 16 (55%) were collected 8─56 days after administration of MR-containing vaccine; of these, nine (56%) were positive by rubella IgM EIA. Compared with measles IgM EIA Virion/Serion and Microimmune, or RT-qPCR as reference, sensitivity of the RDT was improved for both capillary blood (57%, 95% CI: 39%–73%) and oral fluid (38%, 95% CI: 23%–56%). After performing a sensitivity analysis, where indeterminate and discordant visual readings of RDT results were considered as positives, RDT sensitivity further improved, while specificity and PPV were reduced (Table 2 and S2 Table). Among the 16 EIA-concordant cases that had received MR vaccine 8─56 days before specimen collection, eleven cases (69%) were positive by RDT using either capillary blood or oral fluid; five (31%) were positive by RDT using both capillary blood and oral fluid.

Measles RT-qPCR testing (H-gene) of used RDTs from the six cases with throat swabs positive by measles RT-qPCR (N-gene) showed that one (17%) RDT using capillary blood was positive compared to four (67%) RDTs using oral fluid. Three of the six measles cases positive by RT-qPCR using throat swabs had their products sent for genotyping, and B3 (n = 2) and D8 (n = 1) measles genotypes were identified. The same genotypes were obtained by N-gene sequencing of amplicons from two RDTs used for testing oral fluid (B3, n = 1; D8, n = 1).

### Knowledge, attitudes, and practices about measles RDT use

A total of 178 staff involved with the management of measles in the selected clinics and associated health districts responded to the KAP survey at the end of the study (May-June 2020). The largest proportions of respondents were medical officers (46%), followed by laboratory technicians (14%), district health inspectors (12%), and paramedics/medical assistants (10%), with an average of 9 years in their current position (Table 3). Almost all (92%) had previous rapid test experience, most frequently for HIV (82%), pregnancy (76%), or dengue (66%); experience with hepatitis B/C, syphilis, leptospirosis, chikungunya, or malaria rapid tests ranged from 6%-18%. Respondents most frequently reported receiving the formal training on measles RDT use conducted in August 2019 (36%), followed by the cascade training led by district or clinic staff (31%). Among the training materials provided, the standard operating procedure (94%), laminated flow chart (90%), training slides (89%), and demonstration videos (89%) were reported to be the most helpful.

**Table 3:** Survey responses on knowledge, attitudes, and practices about measles rapid diagnostic test (RDT) use at the end of the evaluation period, Malaysia, May-June 2020.

Characteristic	N	%
Respondent role (n = 178)		
Medical officer	82	46
Laboratory technician	25	14
District health inspector	21	12
Paramedic or medical assistant	17	10
Nurse	15	8
Other	18	10
Previous experience with RDT use (n = 178)	164	92
HIV	146	82
Urine pregnancy	136	76
Dengue	117	66
Other	125	70
Duration of experience	**Average(years)**	
In current position (n = 178)	9	
In MOH service (n = 175)	10	
With RDT use (n = 169)	7	
**Training** (n = 178)	**N**	**%**
Type of training received		
Formal training	64	36
Cascade training (called “echo” locally)	55	31
During MOH supervisory visits	18	10
Self-training using provided materials	10	6
Not trained	31	17
Training materials rated very or somewhat helpful		
Standard operating procedure	166	93
Formal/cascade training	164	92
Laminated flow chart	159	89
Demonstration videos	159	89
Training slides	158	89
Post-training quiz	146	82
Monitoring visits	142	80
Online repository with materials	125	70
**Knowledge**	**N**	**%**
Average quiz score on RDT use by type of training received*	178	80
Formal training	64	88
During MOH supervisory visits	18	83
Cascade training	55	78
Self-training using provided materials	10	61
Not trained	31	68
**Practice**	**N**	**%**
Reported reading RDT result at 20 minutes (n = 67)	56	84
Steps in RDT use rated easy or moderate (n = 114)		
Collecting specimens		
Capillary blood	113	99
Oral fluid	100	88
Venous blood	95	83
Throat swab	97	85
Processing specimens		
Capillary blood	109	96
Oral fluid	103	90
Venous blood	91	80
Performing the RDT	111	97
Direct visual reading of RDT	111	97
Shipping RDT and specimens	96	84
Reporting RDT results	106	93
Reported malfunction of test equipment (n = 114)		
Oral fluid collector	3	3
Measles RDT	6	5
Always/often used venous blood over capillary blood for measles RDT (n = 115)	24	21
Frequency of measles RDT results given to patients/caregivers		
(n = 165)		
Always/often	68	41
Sometimes/rarely	32	19
Never	32	19
Not applicable	33	20
**Attitude**	**N**	**%**
Opinion about accuracy of measles RDT (n = 165)		
More accurate than measles laboratory tests	7	4
Similar accuracy to measles laboratory tests	64	39
Less accurate than measles laboratory tests	52	32
Opinions on patients’/caregivers’ most preferred test (n = 164)		
Capillary blood & RDT	91	56
Oral fluid & RDT	53	32
Venous blood & laboratory test	37	23
Opinion about effect of measles RDT on work burden during study		
(n = 165)		
Benefit outweighs added work burden	89	54
Measles RDTs create too much added work burden	37	22
No opinion	39	24
Opinion about where to administer RDTs in Malaysia (n = 167)		
In all health facilities	114	68
At district level and used during outbreaks or as needed	50	30
In higher level health centers/hospitals or central level as needed	18	11
Should not be available in Malaysia	2	1
No opinion	3	2
Opinion on improvements needed before RDT introduction (n = 165)		
RDT supply management (kit, buffer)	76	46
Clinic flow for RDT	73	44
Recording and reporting of RDT results	68	41
Monitoring and feedback on RDT results	64	39
RDT design and performance	61	37
Public health response to RDT results	42	25
Feedback of results to caregivers	39	24
Shipping of RDT and specimens	37	22
Preferred way of reporting RDT results (n = 166)		
Write RDT result on laboratory request form	76	46
Enter RDT result in electronic case notification (e-notifikasi)	114	69
Send picture of RDT result by email or Whatsapp	64	39
Call or text district health office	35	21
No opinion	12	7
Opinion on overall impact of RDT on surveillance program (n = 167)		
Positive	78	47
Some positive and some negative	79	47
Negative	2	1
Don’t know	8	5
Opinion on effect of RDT results on public health response (n = 166)		
Decrease time between case detection and response	97	58
Increase time between case detection and response	18	11
Decrease extent of public health response	61	37
Increase extent of public health response	46	28
Don’t know	12	7

*This was an average quiz score for 10 questions that were the same between the August 2019 post-training test and May-June 2020 KAP survey, including questions on the correct procedures for specimen collection and processing, running the RDTs, and interpretation of RDT results, including several pictures. The average quiz score for the August 2019 post-training test was 91% (n = 224).

Knowledge on RDT use was high among KAP respondents assessed during May–June 2020, with an average knowledge quiz score of 80%. Knowledge was the highest among those who received the formal training (average quiz score: 88%), followed by MOH supervisory visits (83%), and cascade training (78%); 32% of respondents reported never directly using the measles RDT during the study, and quiz scores were generally higher among those who used >5 tests (86%) than those who reported using none (72%). By comparison, the average quiz score using the same questions at the end of the formal training in August 2019 (n = 224) was 91%. Of the ten quiz questions, the percentage of correct responses ranged 78%–97% for seven questions and for the other three questions, correct responses were: 71% for waiting 20 minutes after adding the specimen before reading the RDT, 62% using capillary blood and oral fluid with the RDT, and only 46% for correctly interpreting photographic images of a weak positive RDT (while 44% of staff called the test result “indeterminate”).

In terms of reported practices among staff that used the measles RDT, 84% reported waiting 20 minutes after adding the specimen before reading the test, and 21% reported always or often using venous blood instead of capillary blood in the measles RDT. Among all specimen types, collection and processing were judged to be “easy” to “moderate” for capillary blood (99% and 96%, respectively), followed by oral fluid (88% and 90%) and venous blood (83% and 81%). A high majority of staff judged the following tasks to be “easy” or “moderate” in terms of difficulty: running the RDT (97%), reading the RDT results (97%), and reporting RDT results (93%).

Over half (54%) of surveyed staff reported that the benefits of using measles RDTs outweighed the added work during the study; 43% of staff reported thinking that measles RDTs had similar or greater accuracy to measles laboratory-based tests. In addition, 56% of staff reported that they thought using the RDT with capillary blood was the patients’ (or child caregivers’) preferred test for measles confirmation; 32% reported preference for the RDT with oral fluid. Approximately two-thirds (68%) reported that measles RDTs should be used in all health facilities in Malaysia. Various improvements were suggested before nationwide introduction of RDTs, including RDT supply management (46%) and clinic flow (44%) for RDT use. Almost half of the staff surveyed (47%) judged that the introduction of measles RDTs would have a positive impact on surveillance; 58% of staff reported thinking that the availability of measles RDT results would decrease the time between case investigation and public health response.

### Impact of RDT testing on the measles public health response

In the evaluation of the impact of RDT testing and more rapid case confirmation on the public health response for measles cases confirmed by EIA or RDT (n = 26 in pre-RDT and n = 62 in RDT implementation period), the mean number of days until case investigation (1.0 day), contact investigation (3.4 days), active case detection (8.9 days), and mop-up vaccination (11.7 days) were lower during RDT implementation than in the pre-RDT implementation period (2.2, 4.7, 14.0, and 16.4 days, respectively); however, none of the pre-post differences were statistically significant (Table 4). Compared with the 24 cases that only had positive EIA results in the RDT implementation period, the 38 cases with positive RDT results had lower times until active case detection (11.0 vs. 7.6 days, p = 0.03) and mop-up vaccination (14.8 vs. 9.5 days, p = 0.06).

**Table 4:** Mean number of days from measles case notification to public health response (case investigation, contact investigation, active case detection, mop-up vaccination) during pre- rapid diagnostic test (RDT) and RDT implementation periods, among IgM positive cases *.

Survey and indicator	N	Mean	Std Dev	Range	P-value**
**Pre-RDT implementation period** (n = 26 IgM-positive by EIA)					
Days until case investigation	26	2.2	5.2	0–27	-
Days until contact investigation	26	4.7	12.0	0–57	-
Days until active case detection	22	14.0	17.1	1–66	-
Days until mop-up vaccination	21	16.4	17.1	1–66	-
**RDT implementation period** (n = 62 IgM-positive by EIA or RDT: 20 EIA & RDT; 18 RDT-alone; 24 EIA alone)					
Days until case investigation	62	1.0	1.1	0–4	0.25
Days until contact investigation	61	3.4	7.6	0–45	0.55
Days until active case detection	59	8.9	11.9	0–57	0.09
Days until mop-up vaccination	47	11.7	12.6	0–57	0.27

* Measles IgM-positive cases were positive by enzyme immunoassay (EIA) or rapid diagnostic test (RDT, only used in the RDT implementation period). Measles vaccine-associated reactions were included in this analysis because the public health response to a measles vaccine-associated reaction in Malaysia remains the same as for other measles case classifications.

**Assessed by Wilcoxon two-sample test

## Discussion

In this study, we successfully implemented a pilot of measles IgM RDT testing as part of surveillance in 34 health clinics using one-day regional trainings, cascade trainings, and supervisory visits. Agreement between two readers of the RDTs in health clinics was very high (99% for capillary blood and 97% for oral fluid), similar to previously published results [7]. The KAP survey documented that clinic staff had extensive (92%) previous experience with other RDTs, which likely contributed to the success of piloting measles RDT use in clinics in Malaysia. Knowledge was very high (91%) after the formal training and remained high (80%) ≥9 months later among KAP respondents. Although not statistically significant, the duration of time until public response activities were initiated after case detection decreased by ~5 days during RDT implementation, likely related to receiving RDT results at the point-of-care rather than the 4 days targeted to perform an EIA in the laboratory and return results.

Measles elimination is defined as the absence of endemic measles cases in a defined geographical area (e.g., country or region) for a period of at least 12 months or more, in the presence of a well-performing surveillance system [12]. In such settings, it is important to have a very sensitive surveillance system (i.e., >2 per 100,000 non-measles/non-rubella discard rate) to be able to identify small outbreaks and remaining pockets of susceptible individuals [12]. However, assessing RDT performance in a setting close to elimination (very low incidence) proved challenging, especially after targeted MR vaccination campaigns. The primary analysis of RDT performance was based on the current reference standard used by surveillance programs, a positive measles IgM EIA result or RT-qPCR result. The Virion/Serion assay, an indirect assay used for comparison in this study, has a sensitivity of 98% and a specificity of 88% [13, 14], which is sufficiently accurate to detect chains of transmission as required for surveillance but insufficiently accurate on its own to define true cases for such an evaluation, especially where most specimens (79%) are collected within 3 days of rash onset when EIA sensitivity is lower. In elimination settings, RT-qPCR testing is increasingly relied upon to aid measles case classification because a positive result is definitive, and RT-qPCR can detect measles RNA in specimens taken in the first several days after rash onset. In our study, RT-qPCR testing was complimented by testing specimens with discordant EIA and RDT results with a measles capture EIA from Microimmune (92% sensitivity and 97% specificity) [14] to increase the specificity of measles case classification.

The challenge in classifying cases in settings close to measles elimination was highlighted by the high rate of discordant results (28%) found using two established serum IgM EIAs in the study setting; 16 (55%) of the 29 cases with concordant IgM-positive Virion/Serion and Microimmune EIA results received a MR vaccine 8─56 days prior to specimen collection and likely represented vaccine responses. The overall rubella IgM positivity rate among measles IgM positive cases was high (36%), with 88% of these cases immunized with MR-containing vaccine within the 8–56 days preceding specimen collection, suggesting a potential vaccine response [15]. Using the more rigorous definition for a “true positive,” the measles IgM RDT showed good specificity (≥97%), and sensitivity improved by >10% points but was still lower than anticipated. However, these results need to be interpreted with caution given the high proportion of recent vaccinees among the measles IgM EIA or RT-qPCR positive cases and the low RT-qPCR positivity rate. Sensitivity of IgM detection by RDT was lower using oral fluid (38%) compared to capillary blood (57%) specimens. These results were consistent with data from previous studies, with the difference in sensitivity being attributed to lower concentrations of antibodies present in oral fluids than in serum or capillary blood [5, 6]. Overall performance was substantially lower than findings (>90% sensitivity and specificity) from published laboratory studies and recent field trials using the same RDT in measles outbreak settings in India and Uganda, where true measles prevalence was higher [4–7]. The challenges observed in this study of interpreting results for evaluating measles RDT performance highlight the need for more studies in elimination settings using routine diagnostic RT-qPCR testing for case confirmation to fully evaluate the potential role of the RDT.

Best practices from this RDT study implementation include the high engagement from the Malaysia MOH in leading every stage of the project. Regional trainings were highly effective in acquiring the knowledge and practices in RDT processing and performing RDTs. The training materials used different formats and included standard operating procedures, clinic flow charts, and demonstration videos, and were made available on an online shared drive. Based on reported practices, field staff were compliant with implementing established protocols. Finally, and because Malaysia’s measles public health response protocol is well-defined, we were able to evaluate the impact of RDT testing and more timely case confirmation on public health response activities.

Different challenges were faced during study implementation. The decrease in measles incidence between study design and implementation increased enrollment time and limited statistical power. Measles incidence was 62.1 per million population in 2018 and dropped to 14.8 during the evaluation period, in 2020 [9]. In addition, concomitant MR vaccination campaigns and mop-ups may have contributed to discrepant results from RDT, EIA, and RT-qPCR. Second, logistical issues were faced such as difficulty with managing the supply of RDTs from the national level to districts, related to the expiry of the buffer and the need for it to be stored using cold chain. Third, in measles elimination (i.e., low incidence) settings, the interpretation of discrepant results from RDT and EIA can be challenging and resource intense because of the reduced predictive value of IgM EIA results for case classification and the need for RT-qPCR testing to confirm cases. Finally, competing priorities in the country included a circulating vaccine-derived poliovirus (cVDPV) outbreak and the COVID-19 pandemic which at times limited MOH staff availability.

The study has several limitations. The number of confirmed measles cases was low in terms of allowing robust statistical evaluation of RDT sensitivity and public health impact. For the impact evaluation, observation bias may have occurred during the RDT implementation phase that could have resulted in faster public health response, along with the impact of RDT results being available earlier than conventional EIA results; however, in the RDT implementation period, the number of days until active case detection for cases with positive RDT results was significantly lower compared with those with positive EIA results alone. With the current study design, we were unable to evaluate whether RDT use impacted the rate of measles suspect case investigation.

## Conclusions

The feasibility of introducing the measles IgM RDT to surveillance and its potential benefits to public health responses were shown in a country close to measles elimination. The sensitivity of the measles IgM RDT was lower than reported in earlier evaluations and measles endemic settings. This was in part due to the challenges of case confirmation in a low transmission setting. Future fully-powered studies using highly specific IgM assays and systematic RT-qPCR for case confirmation are needed to establish the role of the device in elimination settings.

## Supporting information

S1 Fig Standard operating procedure for malaysia measles IgM rapid diagnostic test (RDT) evaluation. A simplified version of this flow chart including pictures was also provided as a laminated job-aid to use at the point-of-care. NPHL: national public health laboratory; RDT: rapid diagnostic test.
(TIF)

S1 File Evaluation approval from the Malaysian medical research & ethics committee. (PDF)

S1 Table Comparison of the results obtained for 436 specimens with rapid diagnostic test (RDT) for the detection of measles-specific IgM and with measles -specific IgM indirect and capture enzyme immunoassays (EIAs) or with reverse transcription-polymerase chain reaction (RT-qPCR) for viral detection (indeterminates treated as negatives). (DOCX)

S2 Table Comparison of the results obtained for 436 specimens with rapid diagnostic test (RDT) for the detection of measles-specific IgM and with measles -specific IgM indirect and capture enzyme immunoassays (EIAs) or with reverse transcription-polymerase chain reaction (RT-qPCR) for viral detection (indeterminates treated as positives). (DOCX)
