# Supplementary figures and images for: A measles IgM rapid diagnostic test to address challenges with national measles surveillance and response in Malaysia
Source: PLoS One. 2024 Mar 14;19(3):e0298730. doi: 10.1371/journal.pone.0298730 (PMC10939268; doi:10.1371/journal.pone.0298730)

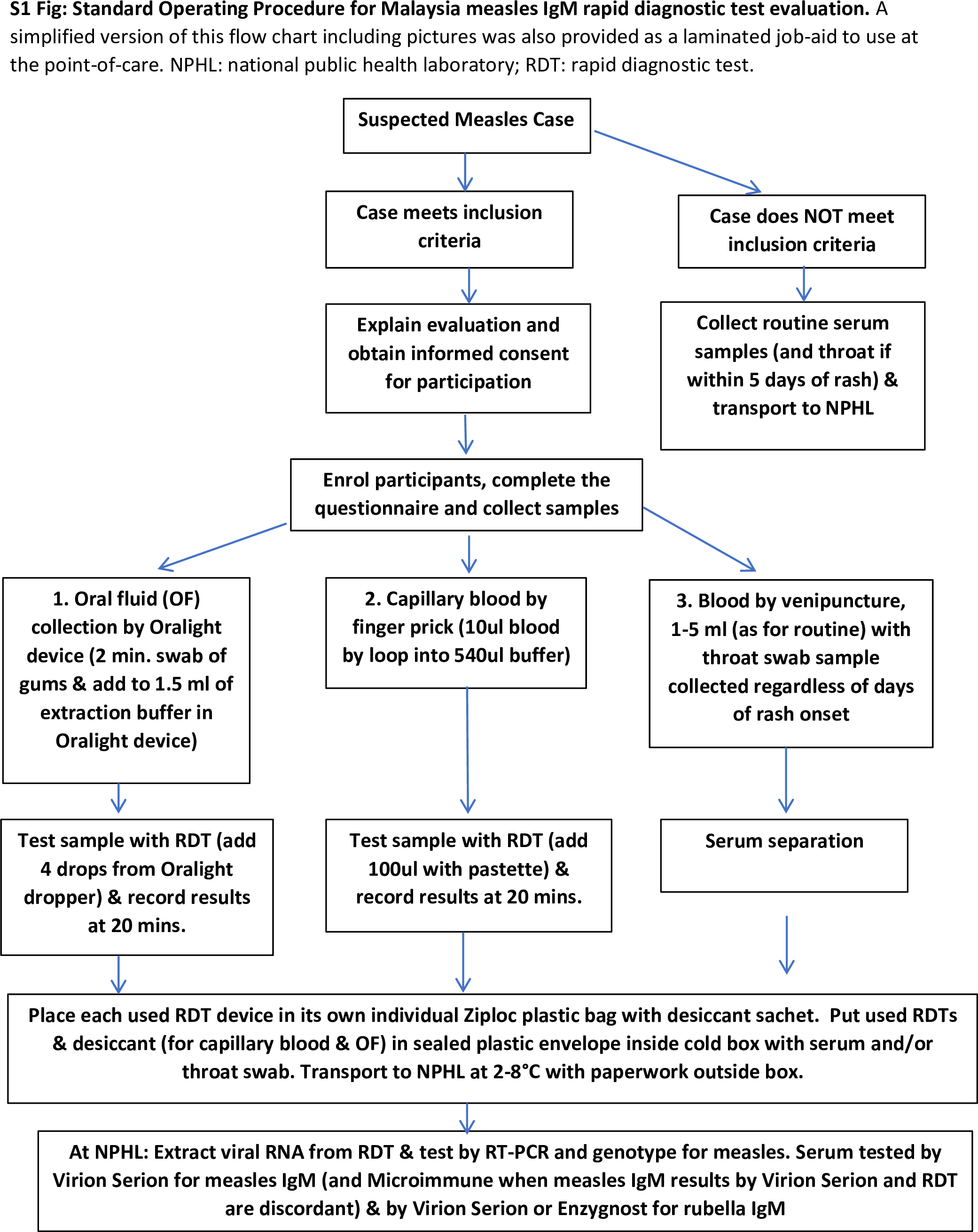

Supplement: S1 Fig — A simplified version of this flow chart including pictures was also provided as a laminated job-aid to use at the point-of-care. NPHL: national public health laboratory; RDT: rapid diagnostic test. (TIF) [file pone.0298730.s001.tif]
