# Supplementary material for: A measles IgM rapid diagnostic test to address challenges with national measles surveillance and response in Malaysia
Source: PLoS One. 2024 Mar 14;19(3):e0298730. doi: 10.1371/journal.pone.0298730 (PMC10939268; doi:10.1371/journal.pone.0298730)
Supplement: S1 File — (PDF) [file pone.0298730.s002.pdf]

JAWATANKUASA ETIKA & PENYELIDIKAN PERUBATAN
(Medical Research & Ethics Committee)
KEMENTERIAN KESIHATAN MALAYSIA
d/a Institut Pengurusan Kesihatan
Jalan Rumah Sakit, Bangsar
59000 Kuala Lumpur

Tel.: 03-2287 4032/2282 0491/2282 9085
03-2282 9082/2282 1402/2282 1449
Faks: 03-2282 0015

Ref:KKM/NIHSEC/P18-1728(13)
Date: 19-October-2018

DR A'AISAH BT SENIN
DISEASE CONTROL DIVISION

Dear Dato'/ Dr/ Sir/ Madam,

ETHICS INITIAL APPROVAL:

NMRR-18-2165-43312 (IIR)
PROTOCOL NO. : N/A
EVALUATING THE FEASIBILITY AND POTENTIAL IMPACT OF INTRODUCING A RAPID DIAGNOSTIC TEST INTO THE NATIONAL SURVEILLANCE PROGRAM IN MALAYSIA TO ENHANCE MEASLES DIAGNOSTIC CAPACITY

This letter is made in reference to the matter above.

2. The Medical Research and Ethics Committee (MREC), Ministry of Health Malaysia (MOH) has provided ethical approval for this study. Please take note that all records and data are to be kept strictly **CONFIDENTIAL** and can only be used for the purpose of this study. All precautions are be taken to maintain data confidentiality. Permission from the District Health Officer / Hospital Administrator/ Hospital Director and all relevant heads of departments /units where the study will be carried out must be obtained prior to the study. You are required to follow and comply with their decision and all other relevant regulations including the Access to the Biological and Benefit Sharing Act 2017.

3. The investigators involved in this study are:

Klinik Kesihatan Bandar Kota Bharu
Dr A'aisah Bt Senin (Principal / Coordinating Investigator)
David Featherstone
Dr Benjamin Dahl
Dr David Brown
Dr Fatanah Ismail
Dr Hani Bt Mat Hussin
Dr Heather Scobie
Dr Jamiatul Aida Binti Md. Sani
Dr Jose Hagan
Dr Mark Papania
Dr Miguel Norman Mulders
Dr Mohamad Izzi Bin Zahari
Dr Noorliza Binti Mohamad Noordin
Dr Paul Rota
Dr Paul Soo
Dr Rozita Bt Ab Rahman
Dr Ying-Ru Lo
Mr James Leonard Goodson
Mr. Selvanesan A/L Sengol
Ms Lenesha Adele Warrener
Ms Zirwatul Adilah Binti Aziz
Varja Grabovac

Klinik Kesihatan Bangi
Dr A'aisah Bt Senin (Principal / Coordinating Investigator)
David Featherstone
Dr Benjamin Dahl
Dr David Brown
Dr Fatanah Ismail
Dr Hani Bt Mat Hussin
Dr Heather Scobie
Dr Jamiatul Aida Binti Md. Sani
Dr Jose Hagan
Dr Mark Papania
Dr Miguel Norman Mulders
Dr Mohamad Izzi Bin Zahari
Dr Noorliza Binti Mohamad Noordin
Dr Paul Rota
Dr Paul Soo
Dr Rozita Bt Ab Rahman
Dr Ying-Ru Lo
Mr James Leonard Goodson
Mr. Selvanesan A/L Sengol
Ms Lenesha Adele Warrener
Ms Zirwatul Adilah Binti Aziz
Varja Grabovac

Klinik Kesihatan Greentown
Dr A'aisah Bt Senin (Principal / Coordinating Investigator)
David Featherstone
Dr Benjamin Dahl
Dr David Brown
Dr Fatanah Ismail
Dr Hani Bt Mat Hussin
Dr Heather Scobie
Dr Jamiatul Aida Binti Md. Sani
Dr Jose Hagan
Dr Mark Papania
Dr Miguel Norman Mulders
Dr Mohamad Izzi Bin Zahari
Dr Noorliza Binti Mohamad Noordin
Dr Paul Rota
Dr Paul Soo
Dr Rozita Bt Ab Rahman
Dr Ying-Ru Lo
Mr James Leonard Goodson
Mr. Selvanesan A/L Sengol
Ms Lenesha Adele Warrener
Ms Zirwatul Adilah Binti Aziz
Varja Grabovac

Klinik Kesihatan Kuala Lumpur
Dr A'aisah Bt Senin (Principal / Coordinating Investigator)
David Featherstone
Dr Benjamin Dahl
Dr David Brown
Dr Fatanah Ismail
Dr Hani Bt Mat Hussin
Dr Heather Scobie
Dr Jamiatul Aida Binti Md. Sani
Dr Jose Hagan
Dr Mark Papania
Dr Miguel Norman Mulders

Dr Mohamad Izzi Bin Zahari
Dr Noorliza Binti Mohamad Noordin
Dr Paul Rota
Dr Paul Soo
Dr Rozita Bt Ab Rahman
Dr Ying-Ru Lo
Mr James Leonard Goodson
Mr. Selvanesan A/L Sengol
Ms Lenesha Adele Warrener
Ms Zirwatul Adilah Binti Aziz
Varja Grabovac

<u>Klinik Kesihatan Kuala Rompin</u>
Dr A'aisah Bt Senin (Principal / Coordinating Investigator)
David Featherstone
Dr Benjamin Dahl
Dr David Brown
Dr Fatanah Ismail
Dr Hani Bt Mat Hussin
Dr Heather Scobie
Dr Jamiatul Aida Binti Md. Sani
Dr Jose Hagan
Dr Mark Papania
Dr Miguel Norman Mulders
Dr Mohamad Izzi Bin Zahari
Dr Noorliza Binti Mohamad Noordin
Dr Paul Rota
Dr Paul Soo
Dr Rozita Bt Ab Rahman
Dr Ying-Ru Lo
Mr James Leonard Goodson
Mr. Selvanesan A/L Sengol
Ms Lenesha Adele Warrener
Ms Zirwatul Adilah Binti Aziz
Varja Grabovac

<u>Klinik Kesihatan Maran</u>
Dr A'aisah Bt Senin (Principal / Coordinating Investigator)
David Featherstone
Dr Benjamin Dahl
Dr David Brown
Dr Fatanah Ismail
Dr Hani Bt Mat Hussin
Dr Heather Scobie
Dr Jamiatul Aida Binti Md. Sani
Dr Jose Hagan
Dr Mark Papania
Dr Miguel Norman Mulders
Dr Mohamad Izzi Bin Zahari
Dr Noorliza Binti Mohamad Noordin
Dr Paul Rota
Dr Paul Soo
Dr Rozita Bt Ab Rahman
Dr Ying-Ru Lo
Mr James Leonard Goodson
Mr. Selvanesan A/L Sengol
Ms Lenesha Adele Warrener
Ms Zirwatul Adilah Binti Aziz
Varja Grabovac

Klinik Kesihatan Masjid Tanah
Dr A'aisah Bt Senin (Principal / Coordinating Investigator)
David Featherstone
Dr Benjamin Dahl
Dr David Brown
Dr Fatanah Ismail
Dr Hani Bt Mat Hussin
Dr Heather Scobie
Dr Jamiatul Aida Binti Md. Sani
Dr Jose Hagan
Dr Mark Papania
Dr Miguel Norman Mulders
Dr Mohamad Izzi Bin Zahari
Dr Noorliza Binti Mohamad Noordin
Dr Paul Rota
Dr Paul Soo
Dr Rozita Bt Ab Rahman
Dr Ying-Ru Lo
Mr James Leonard Goodson
Mr. Selvanesan A/L Sengol
Ms Lenesha Adele Warrener
Ms Zirwatul Adilah Binti Aziz
Varja Grabovac

Klinik Kesihatan Peringgit
Dr A'aisah Bt Senin (Principal / Coordinating Investigator)
David Featherstone
Dr Benjamin Dahl
Dr David Brown
Dr Fatanah Ismail
Dr Hani Bt Mat Hussin
Dr Heather Scobie
Dr Jamiatul Aida Binti Md. Sani
Dr Jose Hagan
Dr Mark Papania
Dr Miguel Norman Mulders
Dr Mohamad Izzi Bin Zahari
Dr Noorliza Binti Mohamad Noordin
Dr Paul Rota
Dr Paul Soo
Dr Rozita Bt Ab Rahman
Dr Ying-Ru Lo
Mr James Leonard Goodson
Mr. Selvanesan A/L Sengol
Ms Lenesha Adele Warrener
Ms Zirwatul Adilah Binti Aziz
Varja Grabovac

Klinik Kesihatan Putrajaya Presint 18
Dr A'aisah Bt Senin (Principal / Coordinating Investigator)
David Featherstone
Dr Benjamin Dahl
Dr David Brown
Dr Fatanah Ismail
Dr Hani Bt Mat Hussin
Dr Heather Scobie
Dr Jamiatul Aida Binti Md. Sani
Dr Jose Hagan
Dr Mark Papania
Dr Miguel Norman Mulders

Dr Mohamad Izzi Bin Zahari
Dr Noorliza Binti Mohamad Noordin
Dr Paul Rota
Dr Paul Soo
Dr Rozita Bt Ab Rahman
Dr Ying-Ru Lo
Mr James Leonard Goodson
Mr. Selvanesan A/L Sengol
Ms Lenesha Adele Warrener
Ms Zirwatul Adilah Binti Aziz
Varja Grabovac

Klinik Kesihatan Seksyen 19
Dr A'aisah Bt Senin (Principal / Coordinating Investigator)
David Featherstone
Dr Benjamin Dahl
Dr David Brown
Dr Fatanah Ismail
Dr Hani Bt Mat Hussin
Dr Heather Scobie
Dr Jamiatul Aida Binti Md. Sani
Dr Jose Hagan
Dr Mark Papania
Dr Miguel Norman Mulders
Dr Mohamad Izzi Bin Zahari
Dr Noorliza Binti Mohamad Noordin
Dr Paul Rota
Dr Paul Soo
Dr Rozita Bt Ab Rahman
Dr Ying-Ru Lo
Mr James Leonard Goodson
Mr. Selvanesan A/L Sengol
Ms Lenesha Adele Warrener
Ms Zirwatul Adilah Binti Aziz
Varja Grabovac

4. The following study documents have been received and reviewed with reference to the above study:

Documents received and reviewed with reference to the above study:

1. Cover letter to MREC (Version 2, dated 16-10-2018)
2. Declaration of Conflict of Interest (COI) (Version 2, dated 16-10-2018)
3. Protocol (Version 2, dated 15-10-2018)
4. Patient Information Sheet (Version 2, dated 16-10-2018)
5. Informed Consent Form (Version 2, dated 16-10-2018)
6. Assent Form (Version 2, dated 16-10-2018)
7. Parental Agreement Form (Version 2, dated 16-10-2018)
8. Questionnaire (Version 1, dated 02-08-2018)
9. Data Collection Form (Version 1, dated 02-08-2018)
10. Follow-up Review Report (Version 2, dated 16-10-2018)
11. IA-HOD-IA, CV and GCP Certification of:
    - Dr A'aisah Bt Senin
    - Dr Noorliza Binti Mohamad Noordin
    - Dr Rozita Bt Ab Rahman
    - Ms Zirwatul Adilah Binti Aziz
12. IA-HOD-IA and CV of:
    - Dr Benjamin Dahl
    - Dr David Brown

- David Featherstone
- Dr Fatanah Ismail
- Dr Hani Bt Mat Hussin
- Dr Heather Scobie
- Dr Jamiatul Aida Binti Md. Sani
- Dr Jose Hagan
- Mr James Leonard Goodson
- Ms Lenesha Adele Warrener
- Dr Mark Papania
- Dr Miguel Norman Mulders
- Dr Mohamad Izzi Bin Zahari
- Dr Paul Rota
- Dr Paul Soo
- Mr. Selvanesan A/L Sengol
- Varja Grabovac
- Dr Ying-Ru Lo

5. Please note that the approval is valid until **18-October-2019**.The following are to be reported upon receiving ethical approval. Required forms can be obtained from the Medical Research Ethics Committee (MREC) website (**http://www.nih.gov.my/mrec**).

i. **Continuing Review Form** has to be submitted to MREC within 1 month (30 days) prior to the expiry of ethical approval.
ii. **Study Final Report** upon study completion to the MREC.
iii. Ethical approval is required in the case of **amendments/ changes** to the **study documents/ study sites/ study team**.
iv. **Applicable for Clinical interventional Studies only:** Report occurrences of **all Serious Adverse Events (SAEs), Suspected Unexpected Serious Adverse Reaction (SUSARs)** and **Protocol Deviation/Violation** at all MREC approved sites to MREC. SAEs are to be reported within 15 calendar days from awareness of event by investigator. Initial report of SUSARs are to be reported as soon as possible but not later than 7 calendar days from awareness of event by investigator, followed by a complete report within 8 additional calendar days.

6. There will be **450** subjects/ patients/ respondents targeted to be enrolled in this study within Malaysia.

7. Please take note that the reference number of this letter must be stated in all future correspondence related to this study to facilitate the administrative processes.

Project Sites:
KLINIK KESIHATAN BANDAR KOTA BHARU
KLINIK KESIHATAN BANGI
KLINIK KESIHATAN GREENTOWN
KLINIK KESIHATAN KUALA LUMPUR
KLINIK KESIHATAN KUALA ROMPIN
KLINIK KESIHATAN MARAN
KLINIK KESIHATAN MASJID TANAH
KLINIK KESIHATAN PERINGGIT
KLINIK KESIHATAN PUTRAJAYA PRESINT 18
KLINIK KESIHATAN SEKSYEN 19

Ref : KKM/NIHSEC/P18-1728(13)

Decision by Medical Research & Ethics Committee:
(√) Approved
(   ) Disapproved

Date of Approval : **19-October-2018**

....

DR. HJH SALINA BINTI ABDUL AZIZ
Chairperson
Medical Research Ethics Committee
Ministry of Health Malaysia
E-mel: mrecsec@nih.gov.my

JAWATANKUASA ETIKA & PENYELIDIKAN PERUBATAN
(*Medical Research & Ethics Committee*)
KEMENTERIAN KESIHATAN MALAYSIA
d/a Kompleks Institut Kesihatan Negara
Blok A, No 1, Jalan Setia Murni U13/52,
Seksyen U13, Bandar Setia Alam,
40170 Shah Alam, Selangor. Tel: 03-3362 8888/8205

Ref : KKM/NIHSEC/ P18-1728 (14)
Date: 17-May-2019

DR A'AISAH BT SENIN
DISEASE CONTROL DIVISION

Dear Sir/ Mdm,

AMENDMENTS FOR STUDY: NMRR-18-2165-43312 (IIR)
Protocol No :
Evaluating the feasibility and potential impact of introducing a rapid diagnostic test into the national surveillance program in Malaysia to enhance measles diagnostic capacity

Your amendment submission dated 19-April-2019 is referred.

2. Amendments of the following have been received and reviewed with reference to the above study:

Documents received and reviewed with reference to the above study:

1. Change of study site to district health office:
    a. Hulu Langat District Healh Office
    b. Petaling District Healh Office
    c. Sepang District Healh Office
    d. Melaka Tengah District Healh Office
    e. Alor Gajah District Healh Office
    f. Jasin District Healh Office
    g. Kinta District Healh Office
    h. Larut, Matang dan Selama District Healh Office
    i. Kota Bharu District Healh Office
    j. Pasir Mas District Healh Office
    k. Tumpat District Healh Office
    l. Rompin District Healh Office
    m. Maran District Healh Office
    n. Kuantan District Healh Office
    o. Titiwangsa District Healh Office
    p. Putrajaya District Healh Office
2. Study Protocol, Version 3 dated 14 March 2018
3. Data Collection Form (DCF)

JAWATANKUASA ETIKA & PENYELIDIKAN PERUBATAN
(*Medical Research & Ethics Committee*)
KEMENTERIAN KESIHATAN MALAYSIA
d/a Kompleks Institut Kesihatan Negara
Blok A, No 1, Jalan Setia Murni U13/52,
Seksyen U13, Bandar Setia Alam,
40170 Shah Alam, Selangor. Tel: 03-3362 8888/8205

The Medical Research & Ethics Committee, Ministry of Health Malaysia operates in accordance to the International Council for Harmonization of Technical Requirement for Pharmaceutical for Human Use (ICH) and Malaysia Guideline for Good Clinical Practice

Comments (if any): NIL

Decision by Medical Research & Ethics Committee:
(√) Approved
(   ) Disapproved

Date of Decision: 17-May-2019

....
DR HJH SALINA BT ABDUL AZIZ
Chairperson
Medical Research & Ethics Committee
Ministry of Health Malaysia
