# Supplementary material for: A measles IgM rapid diagnostic test to address challenges with national measles surveillance and response in Malaysia
Source: PLoS One. 2024 Mar 14;19(3):e0298730. doi: 10.1371/journal.pone.0298730 (PMC10939268; doi:10.1371/journal.pone.0298730)
Supplement: S1 Table — (DOCX) [file pone.0298730.s003.docx]

**S1 Table: Comparison of the results obtained for 436 specimens with rapid diagnostic test (RDT) for the detection of measles-specific IgM and with measles -specific IgM indirect and capture enzyme immunoassays (EIAs) or with reverse transcription-polymerase chain reaction (RT-qPCR) for viral detection (indeterminates as negatives)**

|  | **RDT with capillary blood** | | **Total (missing)** |
| --- | --- | --- | --- |
| **Measles IgM EIA Virion/Serion or RT-qPCR** | **Positive** | **Negative** |  |
| **Positive** | 19 | 25 | 44 (1) |
| **Negative** | 8 | 378 | 386 (5) |
| **Total** | 27 | 403 | 430 (6) |

|  | **RDT with oral fluid** | | **Total (missing)** |
| --- | --- | --- | --- |
| **Measles IgM EIA Virion/Serion or RT-qPCR** | **Positive** | **Negative** |  |
| **Positive** | 11 | 32 | 43 (2) |
| **Negative** | 12 | 363 | 375 (16) |
| **Total** | 23 | 395 | 418 (18) |

|  | **RDT with capillary blood** | | **Total (missing)** |
| --- | --- | --- | --- |
| **Measles IgM EIA Virion/Serion and Measles IgM EIA Microimmune or RT-qPCR** | **Positive** | **Negative** |  |
| **Positive** | 17 | 13 | 30 (0) |
| **Negative** | 10 | 390 | 400 (6) |
| **Total** | 27 | 403 | 430 (6) |

|  | **RDT with oral fluid** | | **Total (missing)** |
| --- | --- | --- | --- |
| **Measles IgM EIA Virion/Serion and Measles IgM EIA Microimmune or RT-qPCR** | **Positive** | **Negative** |  |
| **Positive** | 11 | 18 | 29 (1) |
| **Negative** | 12 | 377 | 389 (17) |
| **Total** | 23 | 395 | 418 (18) |
